# Safety and clinical efficacy of the secretome of stressed peripheral blood mononuclear cells in patients with diabetic foot ulcer—study protocol of the randomized, placebo-controlled, double-blind, multicenter, international phase II clinical trial MARSYAS II

**DOI:** 10.1186/s13063-020-04948-1

**Published:** 2021-01-06

**Authors:** Alfred Gugerell, Ghazaleh Gouya-Lechner, Helmut Hofbauer, Maria Laggner, Franz Trautinger, Gabriele Almer, Anja Peterbauer-Scherb, Marcus Seibold, Wolfram Hoetzenecker, Christiane Dreschl, Michael Mildner, Hendrik Jan Ankersmit

**Affiliations:** 1 Division of Thoracic Surgery, Medical University of Vienna, Waehringer Guertel 18-20, 1090 Vienna, Austria; 2 Aposcience AG, Vienna, Austria; 3 Clinical Department for Skin and Venereal Diseases, Universitaetsklinikum St.Poelten, St. Poelten, Austria; 4 Vienna, Austria; 5 Austrian Red Cross Blood Transfusion Service of Upper Austria, Linz, Austria; 6 Department of Dermatology and Venerology, Kepler University Hospital, Linz, Austria; 7 Department of Surgery, Krankenhaus der Elisabethinen Klagenfurt, Klagenfurt, Austria; 8 Research Division of Biology and Pathobiology of the Skin, Department of Dermatology, Medical University of Vienna, Vienna, Austria

**Keywords:** Diabetic foot ulcer, Secretome-based therapy, Peripheral blood mononuclear cells, Skin, Inflammation, (Impaired) wound healing, Biological, Hydrogel, Clinical trial protocol, Randomized controlled trial

## Abstract

**Background:**

Diabetes and its sequelae such as diabetic foot ulcer are rising health hazards not only in western countries but all over the world. Effective, yet safe treatments are desperately sought for by physicians, healthcare providers, and of course patients.

**Methods/design:**

APOSEC, a novel, innovative drug, is tested in the phase I/II study MARSYAS II, where its efficacy to promote healing of diabetic foot ulcers will be determined. To this end, the cell-free secretome of peripheral blood mononuclear cells (APOSEC) blended with a hydrogel will be applied topically three times weekly for 4 weeks. APOSEC is predominantly effective in hypoxia-induced tissue damages by modulating the immune system and enhancing angiogenesis, whereby its anti-microbial ability and neuro-regenerative capacity will exert further positive effects. In total, 132 patients will be enrolled in the multicenter, randomized, double-blind, placebo-controlled, parallel group, dose-ranging phase I/II study and treated with APOSEC at three dose levels or placebo for 4 weeks, followed by an 8-week follow-up period to evaluate safety and efficacy of the drug. Wound area reduction after 4 weeks of treatment will serve as the primary endpoint.

**Conclusion:**

We consider our study protocol to be suitable to test topically administered APOSEC in patients suffering from diabetic foot ulcers in a clinical phase I/II trial.

**Trial registration:**

EudraCT 2018-001653-27. Registered on 30 July 2019. ClinicalTrials.gov NCT04277598. Registered on 20 February 2020. Title: “A randomized, placebo-controlled, double-blind study to evaluate safety and dose-dependent clinical efficacy of APO-2 at three different doses in patients with diabetic foot ulcer (MARSYAS II)”

**Supplementary Information:**

The online version contains supplementary material available at 10.1186/s13063-020-04948-1.

## Background

According to the World Health Organization (WHO), diabetes is on the rise: the number of persons suffering from diabetes increased from 108 million in 1980 to 422 million in 2014. The global prevalence of diabetes among adults over 18 years of age has risen from 4.7% in 1980 to 8.5% in 2014 [1]. Major complications of diabetes mellitus are the development of foot ulcers and impaired wound healing. The International Working Group on the Diabetic Foot defines a foot ulcer as a full-thickness wound involving the foot or ankle [2]. Non-healing, chronic wounds predominantly remain in early inflammatory stages of wound healing, lacking the controlled synchronization and succession of events that lead to rapid and complete healing. With regard to diabetic ulcers, healing impairment is caused by several intrinsic factors (neuropathy, vascular problems) and extrinsic factors (wound infection, callus formation, and excessive pressure to the site). By definition, this set of predisposing abnormalities in diabetes has been referred to as the “pathogenic triad” of neuropathy, ischemia, and trauma. One abnormality in the wound healing process can lead to another, generating a pathogenic vicious circle in diabetes [3]. Despite better understanding of the underlying disease pathology and improved care of diabetic foot ulcers (DFUs), foot complications are still the most frequent reasons for hospitalization of diabetic patients [4]. Delayed healing of DFU and further related problems (e.g., neuropathy and unnoticed injuries, tissue necrosis, sepsis) are the primary factors leading to lower extremity amputation and death. Moreover, about 50% of patients undergoing foot amputation develop an ulcer in the contralateral limb within 18 months of surgical intervention [5]. Nearly half of all unhealed neuropathic ulcers lead to death within 5 years [6]. In spite of the severe consequences of DFU, no effective medicinal product for treating non-healing ulcers is currently available.

Standard of care: The clinical practice guidelines of the Austrian and German Diabetes Association [7, 8] are in accordance with the British NICE guideline for diabetic foot problems for DFU prevention and management (published: 26 August 2015 nice.org.uk/guidance/ng19) by recommending a multidisciplinary management of diabetic foot complications including measures such as metabolic optimization and treatment of underlying medical diseases, infection control, debridement of devitalized tissue, effective relief from pressure, local wound treatment, therapy of vascular diseases, and education of patients. Adjuvant therapies (e.g., hyperbaric oxygen therapy, cell therapy) are considered to be reserved for patients classified as Wagner stage > 3 in cases, when all treatment possibilities of revascularization have been exhausted and the threat of lower limb amputation is looming. The NICE guideline does not recommend advanced treatment (e.g., using stem cells or tissue transplantation) outside of a clinical trial setting.

MARSYAS II is the first clinical trial to test a secretome-based therapy in DFU. The origins of the tested drug APOSEC go back to stem cell therapy which was investigated in a field of regenerative medicine. This name resumes the expectations of the investigators of the time at the turn of the millennium to be able to regenerate harmed body tissue by applying cells to it that differentiate themselves into new tissue cells. Stem cells were widely used to treat tissue damages with focus on heart failure [9, 10]. However, promising preclinical results often fell far short of expectations in large controlled clinical trials [11]. Meanwhile, the secretome of stem cells showed promising results in tissue repair (including heart, nerves, or skin) indicating that released factors induce regeneration rather than the cells themselves [12–15]. This approach was known as the “paracrine hypothesis” in stem cell research.

The development of APOSEC started with the use of stressed peripheral blood mononuclear cells (PBMCs) instead of stem cells. Based on the findings of Holzinger and colleagues (they used autologous mononuclear cells to treat patients with non-healing skin ulcer [16]), Ankersmit and his research group demonstrated that irradiated mononuclear cells and their secretome exhibit similar therapeutic effects compared to those of stem cells. At first, the PBMCs were used together with their secretome until it was found out that the therapeutic effect is exerted by the secretome alone. In several studies, it could be shown that the secretome is rich in factors preventing tissue damage and promoting tissue repair in heart failure [17, 18]. The advantage of PBMCs compared to stem cells is that they can be isolated more easily and in larger amounts and that they are a waste product of blood transfusion units. Moreover, the use of the secretome without cells but with all favorable factors has decisive virtues over cell-containing products, e.g., in the possibility of viral clearance [19], better storage options, longer shelf life, and easier handling in the application to the patient.

The secretome released by stressed PBMC is an innovative and new biological drug. The drug substance called APOSEC contains a large variety of biomolecule classes, including proteins, extracellular vesicles, anti-microbial peptides, nucleotides, and lipids. It is produced according to good manufacturing practice using buffy coats of strictly selected healthy blood donors and undergoes two orthogonal virus reduction steps to provide a safe high-quality product [19]. The drug product for the investigational medicinal product (IMP) of MARSYAS II is called APO-2, which is APOSEC mixed with hydrogel for topical application.

Preclinical studies showed in experimental small and large animal models that the intravenous application of APOSEC (APO-1, APOSEC in saline solution) attenuates acute and latent myocardial infarction [20, 21], stroke [22], and spinal cord injury [23] and that topical application of APOSEC (APO-2) improves wound healing [24, 25]. Mode of action (MOA) investigations revealed that APOSEC is effective in treating hypoxia-induced tissue damages by employing a large number of different MOAs. APOSEC exerts cytoprotective and regenerative effects. On the one hand, APOSEC prevents the organism from effects of overreaction by the immune system; on the other side, it stimulates certain aspects of tissue regeneration and wound healing. For example, APOSEC induces effects that prevent tissue destruction like platelet inhibition and vasodilation [26] and increased expression of cytoprotective and anti-apoptotic genes in primary cultured human cells [18, 27]. On the other hand, APOSEC also induces effects that promote tissue regeneration and wound healing like enhanced migration of fibroblasts and keratinocytes [27] and increased sprouting of aortic and spinal cord endothelial cells in vitro [23, 25]. Furthermore, it exerts antibacterial activity [28]. No single effective component could be determined in MOA studies [18, 23, 28]. On the contrary, it was shown that the biological activity of individual fractions of APOSEC tested in selected potency and cell-based assays in vitro is significantly inferior compared to that of the entire secretome [25, 29]. This suggests that the effect of APOSEC depends on the synergy of its components. Hence, in the MARSYAS II trial, the multi-faceted disease of DFU will be met by a pleiotropic drug exerting various effects.

## Methods/design

### Study setting

The clinical study protocol (CSP) of the MARSYAS II trial was written in accordance with the Recommendations for Interventional Trials (SPIRIT) guidelines and completing the SPIRIT Checklist (see supplemental file). The study was approved by the Austrian health authorities (Austrian Office for Safety in Health) and the local Austrian ethics committees (Linz and Klagenfurt). A thorough feasibility and site qualification was performed prior to the selection of 16 study sites in 4 countries (Austria, Germany, Poland, and the Czech Republic) to ensure the achievement of adequate participant enrollment to reach the calculated target sample size. The clinical research organization (CRO) FGK Clinical Research GmbH (Munich, Germany) and its subsidiaries in the Czech Republic and Poland will coordinate the centers and are responsible for data management and pharmacovigilance. Any protocol modifications will be timely reported to the respective authority.

The primary objective of the MARSYAS II trial is to determine the dose-response relationship of the clinical efficacy of APO-2 multiple dose administration in patients with diabetic foot ulcer at three different dose levels compared to placebo. The secondary objectives are to evaluate the safety and tolerability of APO-2 at three different doses and to generate data on additional clinical endpoints for the conception of a phase III study.

**Fig. 1:**
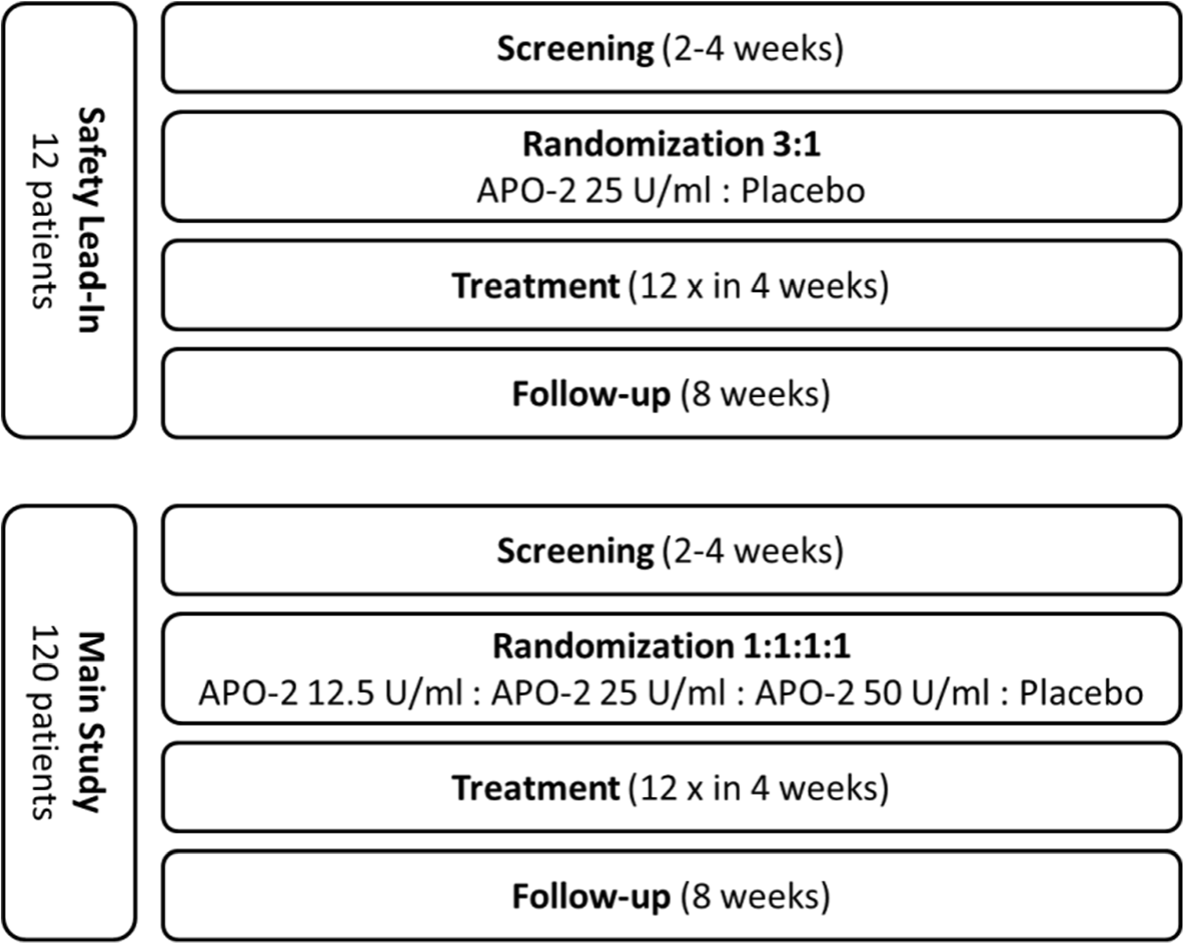
Study design of the MARSYAS II study

The study design is shown in Fig. 1. The partners of the safety lead-in phase are listed in Table 1.

**Table 1:** Partners in the safety lead-in phase of the MARSYAS II study

Sponsor	Aposcience AG
CRO	FGK Clinical Research GmbH
Service provider for IMP storage and distribution	ABF Pharmaceutical Services GmbH
Drug manufacturer	Austrian Red Cross, Blood Transfusion Service for Upper Austria
Safety lead-in	
Study center 1	Kepler Universitaetsklinikum Linz
Study center 2	A.oe. Krankenhaus der Elisabethinen Klagenfurt
Backup study center 1	Fakultní nemocnice u sv. Anny, Brno
Backup study center 2	Fakultní nemocnice Královské Vinohrady, Prague
Backup study center 3	Ústřední vojenská nemocnice - Vojenská fakultní nemocnice, Prague

The study design is based on the results and experiences of the clinical study MARSYAS I, where the topical application of autologous APOSEC was tested in healthy volunteers. The study MARSYAS II is divided into a main study phase (phase IIa, parallel group, four-arm, exploratory trial with equal allocation ratio and superiority testing for secondary endpoints) and a safety lead-in phase (phase I, parallel group, two-arm, exploratory trial with 3:1 allocation ratio) which precedes the main phase and evaluates the safety of the IMP. In the safety lead-in phase, only the medium dose is administered. Between screening and randomization visit, a run-in time period of a minimum of 2 weeks is implemented to observe if the wounds heal when treated with standard of care (SoC) and to include only patients with impaired wound healing.

After the run-in period, the treatment period starts and lasts for 4 weeks, followed by an 8-week follow-up period without application of IMP but with continuation of treatment with SoC.

### Standard study procedures

Before inclusion of a patient into the study, written informed consent will be obtained after disclosure of all aspects of the trial by a physician. The investigator will verify that all inclusion, exclusion, and randomization criteria are met, and all other requirements of the study protocol are fulfilled. The eligibility criteria are listed in Table 2. Patients will be enrolled in various clinical settings, including outpatients from specialized wound management clinics and inpatients from tertiary care setting. The visit schedule is shown in Table 3. On the informed consent form, participants will be asked if they agree to the use of their data: medical findings and personal information will be collected and written down in their personal file at the site; a transfer of the data, in particular to the sponsor, takes place only in pseudonymized and encrypted form. This trial does not involve collecting biological specimens for storage. During their participation in the clinical study, patients will be insured as defined by legal requirements.

**Table 2:** Inclusion/exclusion criteria

Inclusion criteria	
○ Patient is between 18 and 80 years of age	
○ Patients with type I or type II diabetes with a glycosylated hemoglobin (HbA1c) of ≤ 12%, obtained at enrollment or within 30 days prior to study enrollment	
○ Patients who have a wound defined as diabetic foot ulcer present for ≥ 4 weeks	
○ Foot ulcer Wagner grade I–II or ARMSTRONG grade I-A (superficial, non-infected, non-ischemic wound not involving the tendon, capsules, or bone) or II-A (non-infected, non-ischemic wound penetrating to tendon or capsule but not to the bone or joint)	
○ Estimated foot ulcer surface area between ≥ 1 and ≤ 8 cm^2^ as measured at the day of randomization assessed using the eKARE imaging and measurement device	
○ A patient with more than one diabetic foot ulcer may be included in the study but only one ulcer will be selected for the investigational treatment based on investigator judgment as far as the ulcer meets the inclusion criteria (the largest ulcer fitting the inclusion criteria will be selected as index ulcer)	
○ Wound area has not changed by more than 30% between screening visit and randomization visit (at least 14 days)	
○ Adequate arterial blood perfusion (ABI [ankle brachial index] between 0.7 and 1.3 [the highest ABI measured value will be used as a reference], or toe pressure > 50 mmHg, or tcPO2 > 40 mmHg) within the past 6 months	
○ Patient must adhere to off-loading of the ulcer area (in mobile patients, adherence to off-loading footwear during the study is mandatory)	
○ Patient is able to give written informed consent prior to study start and to comply with the study requirements	
○ Women of childbearing potential agree using adequate birth control methods during the study	
Exclusion criteria	
○ History of anaphylaxis, known hypersensitivity to sodium alginate, propylene glycol, methylene blue, or chicken egg	
○ Target ulcer is over a deformity (such as Charcot deformity) that interferes with off-loading based on investigator’s opinion	
○ Index wound duration of > 52 weeks without intermittent healing	
○ Clinical evidence of ulcer bed infection or patients requiring intravenous (IV) antibiotics to treat the index wound infection at time of randomization	
○ Current evidence of osteomyelitis, cellulitis, or other evidence of infection including pus drainage from the wound site, or documented history of osteomyelitis at the target wound location during the 6 months preceding the screening visit	
○ Major uncontrolled medical disorder(s) such as severe uncontrolled leg edema, concurrent medication, or other issues that render the patient unsuitable for participation in the study, including but not limited to comorbid condition with an estimated life expectancy of ≤ 12 months, hemoglobin A1c (Hba1c) > 12% at screening, patients on dialysis, patients with severe pulmonary (requiring home oxygen, uncontrolled COPD Gold III/ IV) or cardiovascular conditions (heart failure NYHA IV, uncontrolled hypertension systolic BP by repeated measurement > 180 mmHg)	
○ Raynaud disease or any other severe peripheral microvascular disease, current diagnosis of vasculitis, or current diagnosis of claudication	
○ Dermatologic comorbid disease, history of systemic lupus erythematosus with elevated anti-DNA antibody titers, Buerger’s disease (thromboangiitis obliterans)	
○ Patient currently treated for an active malignant disease or prior diagnosis of an active malignant disease who is disease free for less than 1 year. Treatment with anticancer therapy (chemotherapy, immunotherapy, radiotherapy, targeted therapy, or gene therapy) within 3 months before the first administration of the investigational product or at any time during the study	
○ Patient with a history of malignancy within the wound; history of radiation therapy to the wound region	
○ Patients who have undergone wound treatments with growth factors, dermal substitutes, or other biological therapies within the last 30 days or during the study	
○ Patients who received oral or parenteral corticosteroids, immunosuppressants, or cytotoxic agents within 30 days preceding the first study drug administration, or plan to use these medications during the study period	
○ Patients who are pregnant or breastfeeding	
○ Mental condition rendering the patient (or the patient’s legally acceptable representative[s]) unable to understand the nature, scope, and possible consequences of the study	
○ Patients who are incarcerated, including prisoners or patients compulsorily detained for treatment of either a psychiatric or physical (e.g., infectious disease) illness	
○ Therapy with another investigational agent within 30 days of screening, or during the study	
○ Patients who are considered by the investigator to have a significant disease, which can impact the study; patients who are considered not suitable for the study by the investigator	
○ Employee at the study site, spouse/partner or relative of any study staff (e.g., investigator, sub-investigators, or study nurse), or relationship to the sponsor	
Prohibited medication	
○ Oral or parenteral corticosteroids of > 20 mg/week within the past 4 weeks before the screening and during the course of the study (intranasal or inhaled steroids for allergies/asthma or COPD are allowed)	
○ Immunosuppressive or cytotoxic agents within 30 days preceding the first study drug administration and during the study period	
○ Wound treatments with growth factors, dermal substitutes, or other topical biological therapies within the last 30 days of screening and during the study duration

**Table 3:** Visit and treatment schedule

Periods	Enrollment/screening	Allocation	Active treatment	Follow-up/close-out			
Visits	Visit 1	Visit 2	Visit 3 to visit 13	14 (EOT)	15	16	17
Days from randomization visit	Days -14 to -28 (prior to active treatment)	Day 0	Day 1 to day 28	Day 30 (± 5 days)	Day 44 (± 2 days)	Day 56 (± 2 days)	Day 84 (± 5 days)
**Enrollment**							
**Informed consent**	X						
**Inclusion/exclusion criteria**	X	X					
**Demography**	X						
**Medical history**	X						
**Concomitant medication**	X	X	X	X	X	X	X
**Interventions**							
**Randomization**		X					
**IMP application (verum)**		X	X				
**IMP application (placebo)**		X	X				
**Assessments**							
**Physical examination and vital signs**	X	X		X			X
**Hematology**^a^	X						X
**Serum chemistry**^b^	X						X
**Urine analysis**^c^	X						X
**Urine pregnancy test**	X	X		X			
**Wound assessment**	X	X	X	X	X	X	X
**Assessment of wound closure**	X	X	X	X	X	X	X
**Wound size measurement (eKare inSight®)**	X	X	X	X	X	X	X
**Standard of care for wound management**	X	X	X	X	X	X	X
**VAS—score for pain**	X	X	X	X	X	X	X
**Neurological assessment foot**		X		X			X
**Chronic wound quality of life questionnaire**		X		X			X
**Recording adverse events**		X	X	X	X	X	X

^a^Erythrocytes/leucocytes/hemoglobin, hematocrit, thrombocytes, MCV, MCH, and MCHC

^b^Sodium, potassium, total protein, albumin, BUN, creatinine, glucose, Hba1c, AST, ALT, GGT, total bilirubin, and CRP

^c^Urine dip stick: pH, nitrite, glucose, leucocyte, ketone, bilirubin, protein, and blood

In the safety lead-in period, a minimum of 12 patients will be enrolled and randomly assigned (3 active: 1 placebo) to receive the medium dose of APO-2, (25 U/ml, 9 patients) or placebo (3 patients). Randomization is done by computer-generated random numbers, and the information is only given to the pharmacist who prepares the IMP. Safety assessments of patients in the safety lead-in period will include recording of adverse events (AEs), use of concomitant medications, and wound size measurement. Immediately after the last randomized patient reaches the end of treatment with IMP, safety and efficacy data of the safety lead-in period will be reviewed by an independent Data and Safety Monitoring Board (DSMB). During this evaluation, study enrollment will be paused and only the active patients will be followed up. The DSMB board consists of three independent experts in the field of clinical wound care and one statistician.

After a review of the safety results, the DSMB will make a recommendation as to whether or not the main study may start as designed (i.e., randomized, double-blind, parallel group). The DSMB may also recommend the inclusion of additional patients in the safety lead-in period prior to initiation of the main study. After the DSMB decision, the patients in the safety lead-in period will continue to be followed as per protocol completing the 8-week follow-up period after the last administration of the investigational product. Besides the DSMB, no other interim analyses or formal stopping rules are intended.

For the main study, eligible patients will be stratified by wound size (at least one third of patients are required to have wound size > 4 cm^2^) and randomly assigned to 1 of 4 treatment groups (low dose, medium dose, high dose, or placebo).

### Screening/inclusion of patients

At the screening visit (visit 1), all patients will be treated according to the standard of care. SoC as described in this protocol follows general guidance and will be carefully applied by all study sites, and all patients have to wear off-loading footwear throughout the whole study. Particularly in the performance of wound cleaning and debridement, the investigators receive additional guidance in the investigator brochure and a training during the site initiation visit. A run-in period of at least 2 weeks will allow assessments of wound area reduction based on optimized SoC. The change of wound area must not exceed 30% between screening visit and randomization visit (14–28 days) to ensure only hard-to-heal DFUs are included into the study. Wound imaging and wound size measurement will be performed using the eKare inSight wound imaging (eKare, Fairfax, VA, USA) at the site level. Data are uploaded to a data management platform and centrally adjudicated by two blinded evaluators. Data for the primary efficacy endpoint will be calculated based on the data generated by the central adjudication assessment. An electronic case report form (eCRF) will be used for this study, and entries and corrections will be performed only by study site staff authorized by the investigator. After all eCRF pages have been reviewed by a monitor and all queries resolved, a final review will be performed by the data review team consisting of the monitors, the data manager, and the medical data reviewer, before database lock during a blinded data review meeting.

A special section is designated to adverse events (AEs) in the eCRF. All AEs will be reported within the annual Development Safety Update Report (DSUR). In the case of a serious AE (SAE), the investigator must complete the SAE report form, the automated eCRF reporting system forwards the SAE report to the drug safety department at the CRO, and the sponsor is informed. SAEs and SUSARs will be reported to the EC and the regulatory agencies according to legal requirements.

The monitor will contact and visit the investigator regularly (as defined in the Monitoring Plan) and will be granted access to all source documents needed to verify the entries in the eCRF and other protocol-related documents, provided that patient confidentiality is maintained in agreement with local regulations. There is no Trial Steering Group implemented in that trial as it was considered a low-risk intervention.

### Dosage of the investigational medicinal product

APO-2 will be applied to patients in three different dosages: low dose (12.5 U/ml), medium dose (25 U/ml), or high dose (50 U/ml). In the control group, the patients will be treated with placebo which is the vehicle of APOSEC (the culture medium used for APOSEC production which is processed like APOSEC including viral inactivation but without cells and therefore without the secretome which represents the active ingredient of the verum). 0.5 ml IMP will be applied per cm^2^ wound surface area for each dose group with a maximum of 4 ml gel on an 8-cm^2^ wound. Therefore, the minimum dose applied in this study will consist of 6.3 U/wound/treatment and the maximum dose applied will be 200 U/wound/treatment (Table 4).

**Table 4:** Dosage in MARSYAS II and toxicological studies

						Maximum doses in toxicological studies		
	Concentration [U/ml]	Area dose [U/cm^2^]	Maximum area dose [U/cm^2^]	Dose [U/kg b.w.]	Dose [U/kg b.w.]	Dose [U/kg b.w.]	Dose [U/kg b.w.]	Dose [U/kg b.w.]
		Units per 1 cm^2^	Units per 8 cm^2^	Minimum dose per 1 cm^2^	Maximum dose per 8 cm^2^	Single dose i.v. rats	4 weeks repeated dose i.v. mice	4 week repeated dose s.c. minipigs
Low dose	12.5	6.25	50	0.104	0.833			
Medium dose	25.0	12.5	100	0.208	1.667			
High dose	50.0	25	200	0.417	3.333	500	500	33.3

Minimum dose/wound treatment, 6.25 U/cm^2^. Maximum dose/wound treatment, 200 U/8 cm^2^

*U* Units, *b.w.* body weight, *i.v.* intravenous, *s.c.* subcutaneous

The IMP will be prepared by unblinded pharmacists at the study sites. The information which IMP is dedicated to which patient is generated in the eCRF and can be retrieved by the pharmacist. Clear instructions for preparation based on standard operating procedures and videos are provided by the sponsor. Blinding of the investigator and the patient is assured by the appearances of the IMP (different doses of APO-2 or placebo) which is in each case the same, and the paper-based communication between the sites and the pharmacies as well as the electronic documentation (and the user rights) in the eCRF is strictly regulated. Also, the sponsor and the statisticians remain blinded until the end of the study. Unblinding in the case of an emergency is possible both in the eCRF or paper based (envelopes at the study site) by the investigator or by the pharmacovigilance in case of a suspected unexpected adverse drug reaction.

### Study endpoints

The primary endpoint is wound area reduction after 4 weeks of treatment with APO-2, i.e., wound area at visit 14 (end of treatment visit) compared to visit 2 (randomization). Secondary endpoints are > 50% reduction in wound area after 4 weeks (binary outcome); wound size at baseline and 1, 2, 3, 4, 6, 8, and 12 weeks after the first application of IMP; proportion of patients with complete wound closure during the 12-week follow-up period (100% re-epithelialization of the wound surface with the absence of drainage); time to complete wound closure; recurrence rate of the ulcer during the 12-week follow-up period; clinical assessment of peripheral neuropathy at baseline, 4, and 12 weeks after the first application of IMP; number of patients with local adverse events or serious adverse events (SAEs) with causal relationship to study medication; evaluation of wound pain by visual analogue scale (VAS) at baseline, at every treatment visit, and 4, 6, 8, and 12 weeks after the first application of IMP; and evaluation of quality of life using Wound QoL questionnaire at baseline, 4, and 12 weeks after the first application of IMP.

Patients who discontinue the trial prematurely after having received at least one dose of IMP will be regarded as “early termination,” and the circumstances and reasons of their discontinuation must be documented in the eCRF. All effort should be done to follow-up with the patient visit schedule as per protocol, but at least a complete end-of-treatment examination should be performed.

### Sample size calculation and power

Sample size calculation for the primary efficacy endpoint is based on the assumptions that after 4 weeks, a wound area reduction of 45%, 50%, and 55% (at a dose of 12.5 U/ml, 25 U/ml, and 50 U/ml, respectively) can be achieved compared to 40% reduction in the placebo group (significance level = 0.05 (two-sided); power = 80%).

Based on estimated wound reductions for each group, a variance of mean of 0.003 was calculated. A standard deviation within groups is estimated 0.156 based on previous studies and expert opinion. The calculated minimal number of patients per group is 23, calculated with Nquery Advisor® (STATCON GmbH, Witzenhausen, Germany) [30].

### Statistical analysis

For the preceding safety lead-in part of the study, safety data of 12 randomized patients will be summarized after the 4-week treatment period. These data will be evaluated by the DSMB. Unblinding is not planned but is possible if requested by the DSMB. Safety and efficacy results of the safety lead-in phase will be included in the data pool of the entire clinical study for final analysis (i.e., the safety lead-in phase is an integral part of the entire MARSYAS II study).

The main study is designed to evaluate the dose-response relationship for clinical efficacy of topical APO-2 at three dose levels compared to placebo. The minimum effective dose, which is defined as the smallest dose with a discernible, useful effect, will be determined.

The primary endpoint is the assessment of the dose-related, relative reduction in wound area from baseline to week 4 (continuous endpoint). Analysis of the primary and secondary efficacy variables will be based on the full analysis set (i.e., all patients with a measured wound size at baseline, who received at least one dose of IMP and have data available at the end of treatment visit). Details are defined in a statistical analysis plan. If data are normally distributed, one-way analysis of variance (ANOVA) will be calculated, including a Dunnett multiple comparison post hoc test, to test each of the active doses against placebo. If Gaussian distribution is not given, a non-parametric Kruskal-Wallis test with Dunns post hoc tests will be performed. If at least one of the doses is statistically significant, then the dose-response relationship is established. In addition, a dose-response model will be developed and tested. Three candidate models (linear, quadratic, and logistic) will be fitted to the data and the Akaike Information Criterion will be used to select the best model. Confidence bands for the estimated dose-response curve will be presented. Binary endpoints will be analyzed by displaying the number and percentage of patients in each dose group. Differences among dose groups will be tested using Fisher’s exact test. For the evaluation of the continuous endpoints, data will be analyzed as absolute values and changes from baseline will be represented by basic statistics (mean, standard deviation, minimum, first quartile, median, third quartile, maximum). A detailed description of the reports and tests is specified in a statistical analysis plan.

For the primary endpoint, the last observation carried forward (LOCF) procedure will be applied. If the visit 14 measurement of wound size is missing, the last available post-baseline measurement will be used.

### Protocol deviation

The decision whether a protocol deviation is relevant or not for the exclusion of patients from the statistical data set will be made on a case by case basis in a data review meeting before database lock. Guidance will be provided by a Protocol Deviation Plan. There will be no special criteria for discontinuing or modifying allocated interventions.

## Discussion

The clinical trial MARSYAS II will be the first clinical trial testing the safety and efficacy of allogeneic secretome of white blood cells in patients with DFU. A phase I trial named MARSYAS I testing autologous APOSEC in ten healthy male subjects was already successfully performed and showed no severe or serious adverse events [31]. As the drug tested in MARSYAS I was autologous and some modifications, such as viral depletion steps, were added to the manufacturing process of the allogeneic APOSEC which will be tested in MARSYAS II, the two studies cannot be compared from a regulatory point of view. Therefore, a safety lead-in first in man phase will precede the main study of the clinical trial to observe the safety and tolerability of allogeneic APOSEC. However, based on data obtained by non-clinical pharmacology and toxicology studies and the phase I trial, we believe that the product is safe.

Wound area reduction in clinical trials, if prospectively defined, indicates relevant biological activity and therefore was chosen as the primary endpoint. Secondary endpoints will help planning subsequent pivotal phase III trials where the primary endpoint has to be complete wound closure. Several randomized controlled trials and cohort studies have shown a strong correlation between wound area reduction at 4 weeks and complete wound closure at 12 or 20 weeks. In studies where wound area reduction was used as an endpoint, a predefined > 50% reduction in the initial area was considered clinically relevant [32–34]. Therefore, the proportion of patients showing a > 50% reduction of the initial wound area is proposed as a secondary endpoint [35]. The run-in period of 14 to 28 days is performed to determine if the wounds heal under standard of care including off-loading footwear. This procedure is implemented to include hard-to-heal wounds and to exclude wounds that are just mistreated. In that case, such patients will not be included in our study.

On the basis of experiences in preclinical wound models and the MARSYAS I clinical trial, we believe that the selected doses of 12.5 U/ml, 25 U/ml, and 50 U/ml are safe for topical administration in DFU patients. All three doses have been used in preclinical proof-of-concept studies of wound models where APO-2 promoted wound healing (closure) [24, 25]. Both the low and the medium dose have been used in the clinical phase I trial MARSYAS I daily for 7 days [31]. The selected doses are within a reasonable safety margin relative to the doses used in non-clinical toxicology studies (10 times lower than the highest dose used in toxicological studies, Table 4) [36].

The rationale for the treatment frequency and duration is based on own non-clinical studies and previously published data by Holzinger et al. using similar drug compounds in patients with chronic wound ulcers [16]. Regular assessment of wound size and characteristics during the phase II trial will allow for further dose adaptation in the pivotal phase III trials. We know from pre-study visits and questionnaires answered by the chosen principal investigators that predominantly small wounds occur. Therefore, a stratification is implemented in the main study that a minimum of one third of patients are required to have wound size > 4 cm^2^.

## Trial status

According to the current time plan, the first patient in the safety lead-in phase was enrolled in autumn 2020. The main study is planned to start in Q2 2021. The last patient out is presumably in Q1 2022 after treatment of a minimum of 132 patients in total. On this date, the clinical study protocol version 2 from 6 November 2019 is valid.

## Supplementary Information

**Additional file 1.** SPIRIT 2013 Checklist: Recommended items to address in a clinical trial protocol and related documents

## Data Availability

The final trial dataset will be archived by the sponsor according to local legal requirements. Results of the trial will be available at the EudraCT and the ClinicalTrials.gov database. Furthermore, results shall be published in a scientific journal. The datasets (only pseudonymized data) analyzed during the current study will be available from the corresponding author on reasonable request. Patient data are stored on site and are not freely accessible.

## Abbreviations

AE: Adverse event
ATMP: Advanced therapeutic medicinal products
b.w.: Body weight
CRO: Clinical research organization
CSP: Clinical study protocol
DFU: Diabetic foot ulcer
DSMB: Data and Safety Monitoring Board
DSUR: Development Safety Update Report
EC: Ethical committee
eCRF: Electronic case report form
i.v.: Intravenous
IMP: Investigational medicinal product
MNC: Mononuclear cells
MOA: Mode of action
PBMC: Peripheral blood mononuclear cell
s.c.: Subcutaneous
SAE: Serious adverse event
SoC: Standard of care
SUSAR: Suspected unexpected serious adverse reaction
U: Units
WHO: World Health Organization

## Definitions for APOSEC

APOSEC: Acronym: SECretome of stressed, APOptotic/necroptotic PBMCs
Drug substance: APOSEC (secretome of stressed PBMCs in culture medium)
Drug product: APOSEC concentrate (200 U/ml) in saline = APO-2
Placebo: Culture medium processed as APOSEC without cells (stressed PBMCs)
APO-1: APOSEC for the treatment of internal diseases (e.g., acute myocardial infarction, myocarditis, stroke, spinal cord injury), intravenous application
APO-2: APOSEC for the treatment of wounds, topical application
IMP: Drug product or placebo in hydrogel
